# Selective Inhibition of Aurora Kinase A by AK-01/LY3295668 Attenuates MCC Tumor Growth by Inducing MCC Cell Cycle Arrest and Apoptosis

**DOI:** 10.3390/cancers13153708

**Published:** 2021-07-23

**Authors:** Bhaba K. Das, Aarthi Kannan, Quy Nguyen, Jyoti Gogoi, Haibo Zhao, Ling Gao

**Affiliations:** 1 Southern California Institute for Research and Education, Long Beach, CA 90822, USA; bhaba.das@va.gov (B.K.D.); jyoti.gogoi@va.gov (J.G.); haibo.zhao@va.gov (H.Z.); 2 Veterans Affairs Long Beach Healthcare System, Long Beach, CA 90822, USA; aarthik1@uci.edu; 3 Department of Dermatology, University of California, Irvine, CA 92697, USA; 4 Genomics High Throughput Sequencing Facility, Department of Biological Chemistry, University of California, Irvine, CA 92697, USA; quyhn@uci.edu

**Keywords:** Merkel cell carcinoma, neuroendocrine skin cancer, aurora kinase, AK-01, LY3295668

## Abstract

**Simple Summary:**

Merkel cell carcinoma is a deadly skin cancer with few treatment options. When the tumor has spread, less than 18% of patients survive past five years, and the mortality rate is 3-times higher than melanoma. Cancer immunotherapy is a promising field, harnessing the patient’s immune system to fight cancer and offering hope to many patients. However, ineligibility or resistance to immunotherapy is a critical challenge; half of all MCC patients are ineligible, and many treated patients either stop responding after an initial positive response or don’t respond at all. In this study, we tested a promising drug based on genomic information from MCC patient tumors. We found that it was highly effective in killing MCC cells and MCC tumors grown in mice; we also observed that MCC genetic characteristics partly predicted how well the drug worked. These results provide strong evidence for its potential clinical application in MCC patients.

**Abstract:**

Merkel cell carcinoma (MCC) is an often-lethal skin cancer with increasing incidence and limited treatment options. Although immune checkpoint inhibitors (ICI) have become the standard of care in advanced MCC, 50% of all MCC patients are ineligible for ICIs, and amongst those treated, many patients develop resistance. There is no therapeutic alternative for these patients, highlighting the urgent clinical need for alternative therapeutic strategies. Using patient-derived genetic insights and data generated in our lab, we identified aurora kinase as a promising therapeutic target for MCC. In this study, we examined the efficacy of the recently developed and highly selective AURKA inhibitor, AK-01 (LY3295668), in six patient-derived MCC cell lines and two MCC cell-line-derived xenograft mouse models. We found that AK-01 potently suppresses MCC survival through apoptosis and cell cycle arrest, particularly in MCPyV-negative MCC cells without RB expression. Despite the challenge posed by its short in vivo durability upon discontinuation, the swift and substantial tumor suppression with low toxicity makes AK-01 a strong potential candidate for MCC management, particularly in combination with existing regimens.

## 1. Introduction

Compared to major skin cancers (melanoma, basal cell carcinoma, and squamous cell carcinoma), Merkel cell carcinoma (MCC) is a rare but highly aggressive neuroendocrine cancer of the skin with increasing incidence [1,2]. The disease-associated mortality rate of MCC exceeds that of melanoma, with a five-year survival rate of <18% in advanced diseases [3]. Between 2000–2013, there was a 95% increase in reported cases of MCC, as compared to a 15% increase in all solid tumors, and its incidence has quadrupled during the past 20 years [4]. Merkel cell polyomavirus (MCPyV) [5], ultraviolet (UV) exposure [6], and immuno-suppression [7] are known major risk factors. Although immune checkpoint inhibitors (ICIs) targeting programmed cell death protein one and its ligand (PD1/PD-L1) have become the standard of care for advanced MCC, resistance develops in the majority of treated patients [8,9,10,11]. Importantly, there is no effective therapeutic alternative for these patients or for 50% of all MCC patients who are ineligible for ICIs due to comorbidities [10].

Over the years, efforts to develop targeted therapy for MCC have not been fruitful. Unlike other solid tumors, inhibitors of tyrosine kinases of growth factors and oncogenes have little clinical effect on MCC [2,12,13]. Moreover, the Wnt signaling pathway is not activated in MCC [14]. One of the significant observations from us and others is the consistent activation of the PI3K/Akt/mTOR pathway in MCC that is independent of the MCPyV status [15,16,17,18]. We have demonstrated that inhibition of the PI3K/Akt/mTOR pathway suppressed MCC tumor growth both in preclinical studies and in the patient [18,19,20,21]. Moreover, a clinical trial designed to restore normal p53-related function in MCC is currently underway (NCT03787602) [22].

Published reports hint that cell cycle regulators are potential therapeutic targets in MCC [23]. However, the therapeutic efficacy of FDA-approved CDK4/6 inhibitors in MCC is limited due to frequent loss of RB1 function in MCC either by an inactivating mutation or MCPyV large antigen integration. Of note, aurora kinases (AURK) are a family of serine/threonine kinase (AURKA, AURKB, and AURKC) involved in cell cycle progression, most importantly during mitosis [24]. AURKA is upregulated in a variety of tumors and has been associated with poor prognosis [25,26,27,28,29,30]. Moreover, it has been reported that AURKA drives the evolution of resistance to EGFR inhibitors in lung cancer [31], and the aberrant activation of AURKA contributes to the highly aggressive nature of lymphoproliferative disorders [32]. Most Aurora kinase inhibitors developed are pan-Aurora or Aurora B/C inhibitors [33,34,35,36,37,38]. The most advanced Aurora inhibitor alisertib (MLN8237) has narrow Aurora A selectivity and has demonstrated anti-tumor activities when combined with various drugs in several human cancers [39,40,41]. Recently, a highly Aurora A-selective inhibitor, AK-01/LY3295668, developed by Eli Lilly (Eli Lilly, Indianapolis, IN, USA), has demonstrated over 1000-fold selectivity versus AURKB and has anti-tumor activities in a broad panel of cancer cell lines as well as in animal models [42]. Moreover, AURKA inhibition has synthetic lethality in cancer cells possessing *RB1* mutation [43]. Importantly, AK-01 has demonstrated anti-tumor activities in patients with locally advanced or metastatic solid tumors with a manageable toxicity profile in phase I clinical trials [44]. In relevance to MCC, inactivation of RB1 either by *RB1* mutation or integration of MCPyV genome is commonly detected in MCC [45,46,47]. Additionally, RNA-seq data from patient tumors revealed that cell cycle regulatory genes are the top expressed genes in MCC (data will be presented elsewhere). Notably, a high-throughput drug screen identified aurora kinase as one of the top candidate targets across a panel of six MCC cell lines (data will be presented elsewhere).

We chose the recently developed AK-01 for further in vitro and in vivo investigations. We found that AURKA inhibition by AK-01 has potent anti-MCC activities in three of six MCC cell lines independent of MCPyV status. Moreover, AK-01 retains synthetic lethality in MCPyV-negative cells lacking RB expression. Consistent with published studies, AK-01 induces G2-M cell cycle arrest and apoptosis in MCC cells. Moreover, AK-01 significantly debilitated MCC xenograft tumor growth in vivo. However, the MCC xenograft tumor relapsed upon discontinuation of treatment, and re-treatment with AK-01 failed to repress the tumor growth. This evidence suggests that AK-01, in addition to its potential as a single-agent therapy, has clinical potential in combination with other therapies to maximize anti-tumor activities in advanced MCC.

## 2. Materials and Methods

### 2.1. Critical Reagents and Compounds

Aurora Kinase A (AURKA) specific inhibitor, AK-01, was purchased from Chemietek (Indianapolis, IN, USA). AK-01 stock solution was prepared in sterile DMSO at a final concentration of 10 mM and stored at −80 °C in small aliquots. Primary antibodies to cyclin-B1 (cat. no. 4135) and cleaved poly (ADP-ribose) polymerase (PARP) (cat. no. 9542), as well as horseradish peroxidase (HRP) conjugated secondary antibodies for rabbit (cat. no. 7074) and mouse (cat. no. 7076) were purchased from Cell Signaling Technology (Danvers, MA, USA). Antibodies for histone-3 (cat. no. ab1791) and α-tubulin (cat. no. T6199, clone DMIA) were purchased from Abcam (Cambridge, MA, USA) and Millipore Sigma (St. Louis, MO, USA), respectively. RPMI-1640 and Dulbecco’s Modified Eagle’s Medium (DMEM) were purchased from American Type Culture Collection (ATCC, Manassas, VA, USA). Fetal bovine serum (FBS) and tissue culture supplements were obtained from Atlanta Biologicals (Flowery Branch, GA, USA) and Life Technologies (Houston, TX, USA), respectively. Additional reagents include Radioimmunoprecipitation assay (RIPA) buffer (cat. no. R0278, Millipore Sigma) and enhanced chemiluminescence (ECL) detection reagent (cat. no. WBULS0100, Millipore Sigma).

### 2.2. Generation of MCC Cell Line Derived Xenograft Models in Mice

MCC cell-line derived xenograft mouse models were generated using 6-to-8-week-old immunodeficient NOD/SCID/IL2r-ynull (NSG) mice (Strain #5557; Jackson Laboratory, Bar Harbor, ME, USA) [48,49,50]. Briefly, 2 × 10^7^ MCC cells in logarithmic growth were prepared in Matrigel (cat. no. 354248; Corning Life Sciences, Tewksbury, MA, USA) and subcutaneously inoculated on the right rear flank of each mouse. Palpable tumor growth appeared within 3 to 5 days of inoculation, and a treatment regimen was initiated when all xenografts reached a minimum tumor volume of 100 mm^3^. Tumor-bearing mice were randomized into control and treatment groups (*n* ≥ 4 for each condition), with the treatment group receiving 50 mg/kg AK-01 by oral gavage twice daily (formulated in 100 mM phosphoric acid, pH 2.5–3) and the control group receiving an equivalent dose of the vehicle. All animals were monitored daily, and tumor volume was measured using digital calipers and calculated as *L* × *W*^2^/2, where *L* is the longer dimension (length), and *W* is the shorter dimension (width). All animal experiments were conducted under protocols approved by the Institutional Animal Care and Use Committee (IACUC) at the Veterans Affairs Long Beach Healthcare System (VALBHS), in accordance with laboratory animal care and use guidelines set by the Association for Assessment and Accreditation of Laboratory Animal Care (AAALAC) International.

### 2.3. Cell Culture

Patient-derived MCC cell lines (MCC-3, MCC-5, MCC-9, MCC-16, and MCC-21) were established in our lab under study protocols first approved by the Institutional Review Board (IRB) at the University of Arkansas for Medical Sciences, and are currently maintained under study protocols approved by the VALBHS IRB, in accordance with the Declaration of Helsinki and relevant regulations. MKL-1 is a classical MCPyV-positive MCC cell line [6] that was gifted by Dr. Becker (Department of Dermatology, University Hospital Essen, Essen, Germany). Suspension cultures of MCC cells were maintained in RPMI-1640 medium supplemented with 10% FBS, penicillin-streptomycin (100 U/mL), and L-glutamine (4 mM) at 37 °C in a humidified atmosphere with 5% CO_2_. Cells were fed with fresh complete media every 48 h and split 1:2 weekly to maintain logarithmic growth. Cell lines were authenticated via STR-profiling (Genetica, Burlington, NC, USA), comparing each cell line with its respective primary tumor as described previously [19].

### 2.4. Cell Proliferation and Viability Assay

Cell proliferation and viability were measured by Cell Counting Kit-8 (cat. no. 96992, Millipore Sigma) and trypan blue exclusion staining (cat. no. T8154, Millipore Sigma) per manufacturer’s protocols. In brief, cells were plated at 8 × 10^5^ density per well in 96-well plates, allowed to recover for 4 h, and exposed to serial dilutions of AK-01 (0–10 μM dose range) at 37 °C for 72 h. CCK-8 (10% of culture volume) was added to each well and incubated for an additional 4 h at 37 °C. Absorbance at 450 nm was measured, and half-maximal growth-inhibitory dose (GI_50_) was calculated by setting the mean control absorbance as maximal cell proliferation for each cell line, respectively and using nonlinear regression analysis (GraphPad Prism v6.07, San Diego, CA, USA; “log (inhibitor) vs. normalized response” dose-response equation) to identify the concentration at which 50% of maximal cell proliferation was suppressed. Cell viability upon AK-01 treatment was also assessed by trypan blue exclusion staining. In brief, MCC cells were plated in 6-well plates at 5 × 10^5^ density per well, followed by 4 h recovery and AK-01 treatment at 37 °C for 72 h. Cells were then collected, washed, and stained with trypan blue; live cells in each treatment condition were quantified and are presented as a percentage of DMSO control.

### 2.5. Cell Cycle Analysis by Flow Cytometry

Propidium iodide (PI) staining was used to analyze cell cycle distribution in MCC cells treated with AK-01 or vehicle control. MCC cells seeded at 5 × 10^5^ per well in 6-well plates were treated with 300 nM of AK-01 for 48 h, followed by washing and fixation per manufacturer’s protocol. Upon staining with PI/RNAse staining buffer (cat. no. 550825; BD Pharmingen, San Diego, CA, USA), cell cycle progression was analyzed using the ACEA NovoCyte flow cytometer (Agilent, Santa Clara, CA, USA) and NovoExpress (v1.4.1) software (Agilent, Santa Clara, CA, USA) at the Institute for Immunology flow cytometry core facility (University of California, Irvine, CA, USA).

### 2.6. Immunoblotting

MCC cells treated with vehicle or AK-01 for 24 h or 72 h were harvested and processed for immunoblotting analysis as described previously [19,20,21]. Briefly, whole-cell lysates were prepared with 1× RIPA lysis buffer (cat. no. R0278, Millipore Sigma, Burlington, MA, USA) containing cOmplete EDTA-free Protease Inhibitor Cocktail (cat. no. 4693159001; Roche), incubated on ice for 30 min, and clarified by centrifugation at 14,000 rpm for 15 min at 4 °C. Whole-cell protein lysates (10–30 µg per lane) were resolved by 8%, 10% or 12% Tris-Glycine SDS polyacrylamide gel electrophoresis and transferred onto 0.45 µm PVDF membranes (cat. no. IPVH00010, Millipore Sigma, Burlington, MA, USA) using Trans-Blot^®^ SD Semi-Dry Transfer Cell (cat. no. 1703940, BioRad, Hercules, CA, USA). Membranes were blocked with 5% fat-free milk (cat. no. 170-6404, BioRad, Hercules, CA, USA) in 1× Tris-buffered saline pH 7.4 at room temperature (RT) for 1 h, followed by incubation with specific primary antibodies at 4 °C overnight. After washing in 1× Tris-buffered saline and 0.2% Tween-20 (cat. no.161-0781, BioRad, Hercules, CA, USA), membranes were probed with HRP-conjugated secondary antibodies for 1 h at RT and immunoreactive proteins were visualized on X-ray films (Kodak, Rochester, NY, USA) using the ECL kit (cat. no. WBULS0100, Millipore Sigma) as per manufacturer’s instructions. Alpha-tubulin was used as the loading control, and all data are presented on contiguous lanes.

### 2.7. Statistical Analysis

All measurements were made in triplicate, and all values are represented as mean ± SD or mean ± SEM, as noted in figure legends. Statistical analyses were performed with a Student’s *t*-test or one-way analysis of variance (ANOVA) using GraphPad Prism software (v6.07; San Diego, CA, USA), and *p*-values < 0.05 were considered statistically significant.

## 3. Results

### 3.1. AK-01 Suppresses MCC Cell Proliferation and Viability In Vitro

**Figure 1:**
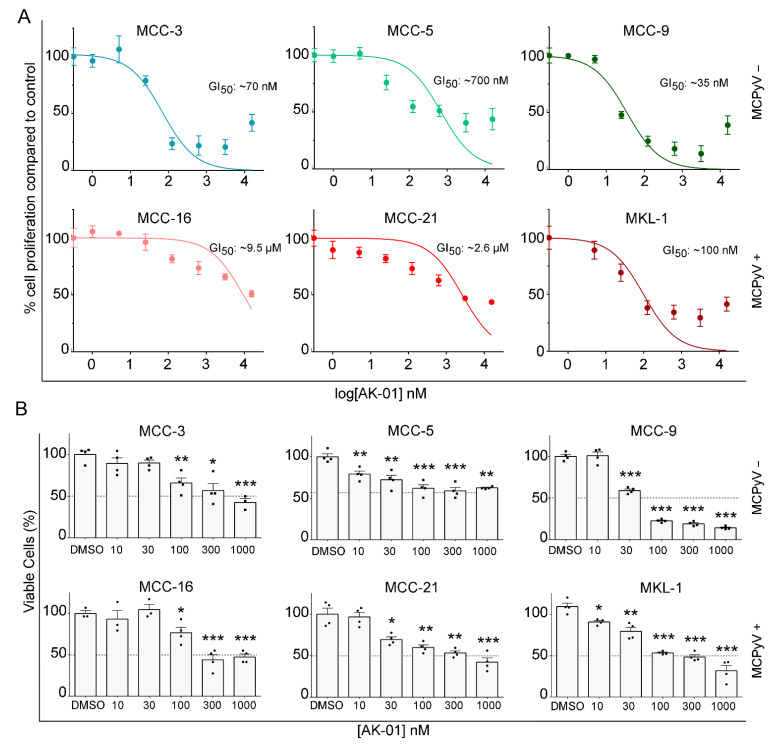
AK-01 attenuates cell viability of MCC cells in vitro. Six MCC cell lines were treated with vehicles (DMSO) of increasing concentrations of AK-01 for 72 h. (**A**) Cell proliferation was assessed by CCK-8 assay with reference maxima (100%) defined as the mean proliferation for vehicle-treated controls. Half maximal growth inhibitory concentration (GI_50_) was calculated by nonlinear regression analysis [GraphPad Prism 6.0, “log (inhibitor) vs. normalized response” dose-response curve equation]. Data presented as mean percent proliferation ± SEM of vehicle control from three experiments (*n* = 6). (**B**) Live cells were quantified by trypan blue dead-cell exclusion staining. Data presented as mean percentage ± SEM of vehicle-treated control (*n* = 3). Dotted line represents 50% cell viability. Experiments were repeated three times. * refers to *p* < 0.05, ** refers to *p* < 0.005, and *** refers to *p* < 0.0005 vs. DMSO-treated cells by paired Student’s *t*-test.

Based on prior reports of anti-tumor effects of AK-01 on other cancers and our high-throughput drug screening data, we examined the effect of AK-01 treatment on a panel of six MCC cell lines (MCPyV-negative cell lines MCC-3, -5, and -9; MCPyV-positive cell lines MCC-16, -21, and MKL-1). MCC cells were exposed to serial concentrations of AK-01 (0–10 μM) for 72 h, and DMSO (vehicle) treated cells served as respective controls for each cell line. Cell viability was measured using CCK-8 cell viability assay as described previously [19]. Half maximal growth inhibitory concentration (GI_50_) was calculated by nonlinear regression analysis using GraphPad Prism v6.07 (“log (inhibitor) vs. normalized response” dose-response curve equation) with average control cell proliferation set as maximal cell proliferation for each cell line, respectively. As depicted in Figure 1A, AK-01 demonstrated potent anti-MCC activities in three out of six cell lines (MCC-3, -9, and MKL-1), whereas MCC-5, -16, and -21 were less responsive. Of the six cell lines, MCC-9 had the lowest GI_50_ of ~35 nM, with MCC-3 being second lowest with ~70 nM.

Cell viability was also evaluated by trypan-blue dead cell exclusion staining (Figure 1B) upon AK-01 treatment. The numbers of live cells were quantified in all conditions, with the control group of each cell line set as the reference maxima (100%) and cells in each treatment group plotted as the percentage of control. As expected, the six cell lines displayed varying levels of AK-01 susceptibility, with MCC-9 and MKL-1 being the most receptive with a 50% reduction in viability observed at ~30 nM and ~100 nM, respectively, which corroborated CCK-8 data. Recent studies have suggested that AURKA inhibition is synthetic lethal in *RB^−/−^* tumors [43,51], prompting us to examine RB expression in six cell lines. MCPyV-negative MCCs commonly have loss-of-function *RB1* mutation, and RB expression is undetectable in those MCC, whereas integration of MCPyV large T antigen in the RB promoter region is frequently found in MCPyV-positive tumor cells [46,47]. RB protein was undetected in two out of three MCPyV-negative cell lines (MCC-3 and -9), whereas all three MCPyV-positive cell lines expressed RB (Figure S1). Notably, AK-01 demonstrated the most potent anti-tumor activities against MCC-3 and MCC-9 and a substantially less potent response against MCC-5, the MCPyV-negative cell line with RB expression. These results hint that *RB1* synthetic lethality was retained by the highly selective AURKA inhibitor AK-01 in MCPyV-negative MCC cell lines. In contrast, RB was expressed in all MCPyV-positive cell lines, with MKL-1 cells being significantly more responsive to AK-01 treatment, implying distinct underlying mechanisms of AURKA-mediated anti-MCC activities in MCPyV-positive MCC.

### 3.2. AK-01 Induces Cell-Cycle Arrest and Apoptosis in MCC Cells In Vitro

**Figure 2:**
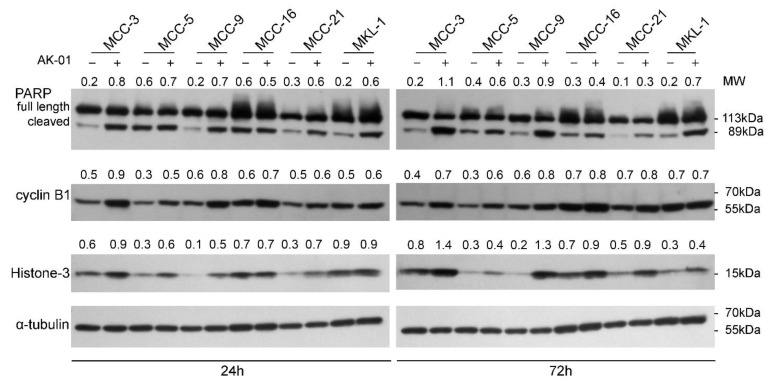
AURKA inhibition causes increased cleaved PARP and accumulation of cyclin-B and histone-3 in MCC cells. Vehicle (DMSO) or AK-01 (300 nM) treated MCC cells were harvested after 24 or 72 h, and 10–30 μg of total cell lysate per lane were resolved in SDS-PAGE followed by immunoblotting using specific antibodies for PARP (total and cleaved), cyclin-B1, H3, and tubulin (loading control). All data represent contiguous lanes from one of three replicate experiments. Densitometry data (ratio of target protein compared to tubulin) was calculated using ImageJ and is presented above each lane of each respective immunoblot. Full western blot images are available in Figures S3–S6. MW, molecular weight; kDa, kilodaltons.

It has been previously shown that AURKA dependent G2-M arrest is associated with an increase in cyclin-B1 and histone-3 accumulation as well as increased apoptosis [35,42,52,53]. To further characterize the effects of AK-01 on MCC cells, we performed immunoblotting analyses of G2-M arrest and apoptosis in six MCC cell lines. Whole-cell protein lysates from MCC cells treated with AK-01 for 24 h or 72 h were probed with specific antibodies, and DMSO-treated cells served as controls. Concurrent with reported findings, both cyclin-B1 and histone-3 levels were higher in AK-01 treated cells compared to controls, particularly in MCPyV-negative cell lines (Figure 2). To assess the apoptotic state of these cells, we also examined the levels of cleaved poly (ADP-ribose) polymerase (PARP). Full-length PARP is associated with cell viability, whereas its cleavage at Asp214 and Gly215 by caspase-3 facilitates cellular disassembly and apoptosis. As shown in Figure 2, AK-01 treatment had a profound impact on PARP cleavage. There was a significant increase in the 89 kDa cleaved carboxy-terminal catalytic domain upon AK-01 treatment compared to controls, and AK-01 treatment-induced apoptosis as suggested by increased cleaved-PARP, more prominent in MCC-3, MCC-9, and MKL-1 cells (Figure 2).

**Figure 3:**
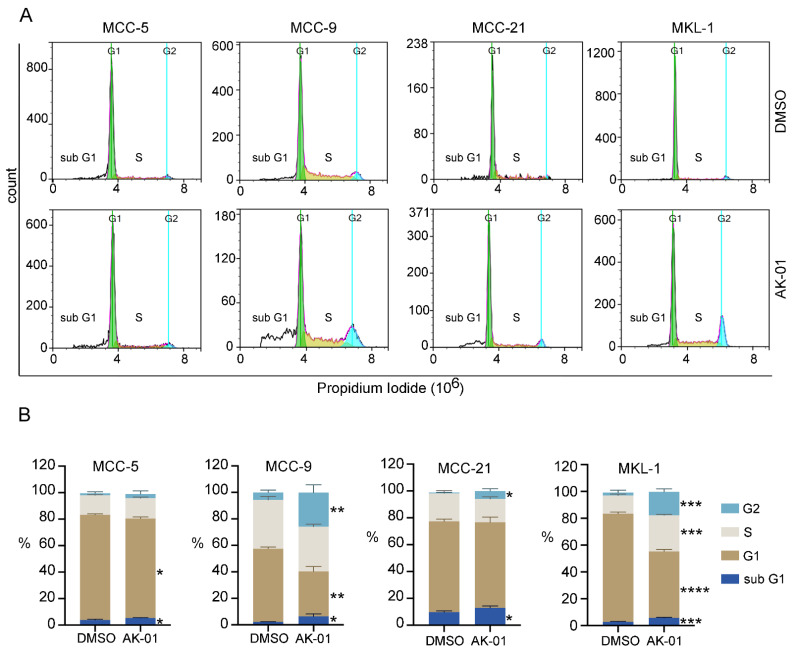
AK-01 induces G2-M cell cycle arrest and apoptosis in MCC cells. MCC cells were treated with vehicle (DMSO) or AK-01 300 nM for 48 h. Cells were then stained with PI (propidium iodide) and analyzed by flow cytometry. DMSO-treated cells served as controls. (**A**) DNA histograms of DMSO (upper panel) and AK-01 (lower panel) treated cells depict a significant increase in sub G1 (white), and G2 (cyan) populations of AK-01 treated MCC cells. (**B**) Percentage histograms depicting the distribution of MCC cell population in cell cycle phases: sub G1 (dark blue), G1 (light brown), S (light gray), and G2 (light blue). Data presented as mean ± SD (*n* = 3). * refers to *p* < 0.05, ** refers to *p* < 0.005, *** refers to *p* < 0.0005, **** refers to *p* < 0.00005 vs. DMSO-treated cells by paired Student’s *t*-test. G1, growth 1 phase; G2, growth 2 phase; S phase, synthesis phase.

To further confirm our above observations, we analyzed cell-cycle progression by flow cytometry in four MCC cell lines upon AK-01 treatment. An increase of cell numbers at the G2 phase upon treatment indicates arrested G2 to M-phase transition, a characteristic phenotype associated with AURKA inhibition. As shown in Figure 3, AK-01 treatment significantly increased cell population in the G2 phase in three cell lines tested (no significance in MCC-5 cells), particularly pronounced in MCC-9 and MKL-1 cells. While cyclin B1 and histone-3 levels in MKL-1 cells were less changed by AK-01 as demonstrated by immunoblotting, a significant increase in sub-G1 population (apoptotic cells) was also observed upon AK-01 treatment, again more prominent in MCC-9 and MKL-1 cells and corroborating cleaved PARP evidence by immunoblotting (Figure 2 and Figure 3). These results provide definitive evidence supporting augmented cell-cycle arrest and heightened apoptosis as the major driver of AK-01′s anti-MCC efficacy.

### 3.3. Aurora Kinase A Inhibition by AK-01 Attenuates MCC Xenograft Tumor Growth

**Figure 4:**
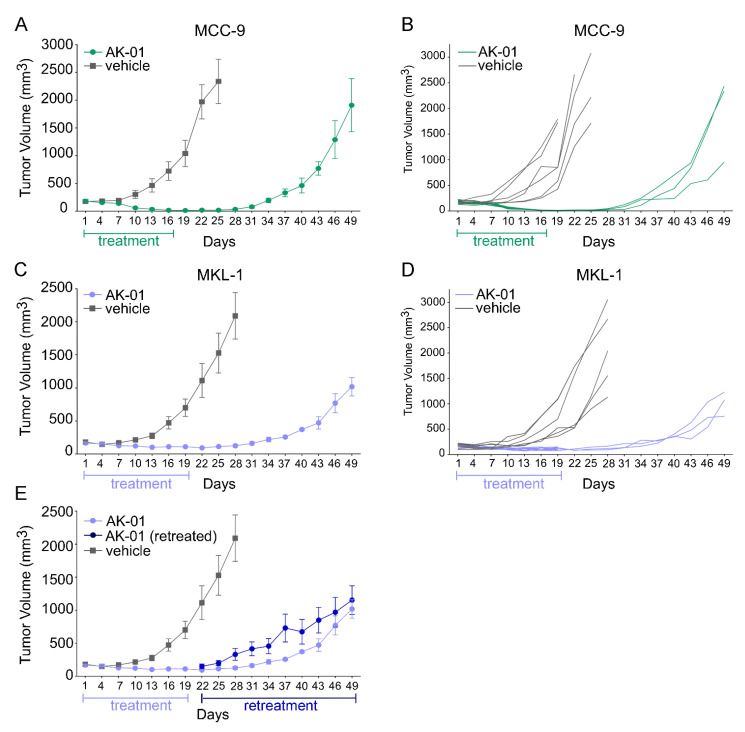
AK-01 mitigates tumor growth of MCC xenografts in NSG mice. NSG mice bearing MCC-9 (**A**,**B**) or MKL-1 (**C**–**E**) xenograft tumors were treated with vehicle or 50 mg/kg AK-01 twice daily by oral gavage for 16 or 19 days, respectively. After randomization, tumor volume was monitored 3× weekly and presented as mean volume ± SEM (**A**,**C**,**E**) or individually (**B**,**D**). In both MCC-9 and MKL-1 xenograft cohorts, three AK-01 treated mice were observed for tumor relapse (days 16–49 and days 19–49, respectively) after discontinuing treatment. For MKL-1, three AK-01 treated mice resumed treatment upon tumor relapse (**E**; retreated mice represented in dark blue).

Hence, these in vitro data prompted us to investigate the efficacy of AK-01 against MCC tumors in vivo. MCC cell-line derived tumor models were generated, and MCC-bearing mice were randomized into control and treatment groups, as described in our earlier work [19] and in Materials and Methods. To simulate the clinical scenario, mice began AK-01 treatment when tumors were established at 100–200 mm^3^. Similar to results from the Phase one clinical trial [44], AK-01 displayed no obvious toxicity as monitored by body weight, activity, and food/water intake (Table S1, Figure S2). In accord with in vitro findings, clear evidence of regression was observed in both MCC-9 as well as MKL-1 derived xenografts. Of the two, MCC-9 was slightly more responsive to the treatment, with tumor volume receding to indiscernible levels within 16 days of treatment. AK-01 treatment was thus discontinued after 16 days of treatment (day 16 post-randomization), and six AK-01 treated mice were sacrificed for histological confirmation. To evaluate the durability of response, three remaining mice were observed for relapse, with xenografts becoming palpable again 12 days after AK-01 discontinuation, and the cohort was sacrificed on day 49 post-randomization (Figure 4A,B). In contrast, though AK-01 significantly repressed MKL-1 xenograft growth, tumors remained discernible after 19 days of treatment. Three mice were sacrificed for histological examination, and the remaining six mice had discontinued treatment and were observed for relapse (Figure 4C,D). All six mice were found to have a relapse within a week of discontinuation of treatment. Surprisingly, three retreated mice had already developed AK-01 resistance and did not respond to further treatment (Figure 4E). Interestingly, the more responsive MCC-9 xenograft was visibly more rampant upon recurrence compared to MKL-1. The underlying mechanism(s) are unclear and warrant further investigation.

## 4. Discussion

Merkel cell carcinoma (MCC) is an aggressive neuroendocrine skin cancer that has quadrupled in incidence over the past 20 years. MCC is far more deadly than melanoma, especially if not detected and treated at an early stage, with a dismal five-year survival rate of <18% in advanced disease. Between 2000–2013, there was a 95% increase in reported cases of MCC, as compared to a 15% increase in all solid tumors. MCC predominantly and disproportionately affects white males older than 65. In addition to MCPyV, UV is the other suggested etiology for MCC without detectable virus. Recent PD1/PD-L1 checkpoint inhibitors have demonstrated durable response and clinical benefits in MCC patients; however, a significant portion of MCC patients either do not respond or acquire resistance, eventually succumbing to their diseases. Yet, to date, there is no effective targeted therapy for MCC approved by the US Food and Drug Administration (FDA). Unlike other solid tumors, activating mutations in tyrosine kinases of growth factor receptors are not detected in MCC, and the MAPK signaling pathway is not constitutively activated in MCC [1,14]. Consistently, a pilot clinical trial failed to demonstrate the clinical benefits of the tyrosine kinase inhibitor Gleevec in advanced MCC patients [54]. We, and others, have reported that the aberrant activation of the phosphatidylinositol-3-kinase (PI3K) pathway is detected in more than 80% of MCC. Inhibition of the PI3K/mTOR pathway has demonstrated therapeutic efficacy in MCC mouse models and in the clinic [19,20,55]. Recently, inhibitors of histone deacetylases (HDACs), a key epigenetic modulator of gene transcription, have been shown to induce cell cycle arrest and apoptosis in MCC cells [23]. Currently, there are several MCC clinical trials with potential targeted therapies as monotherapy or in combination with immunotherapy (NCT04393753, NCT04261855, NCT04521413, NCT03787602).

Cancer is characterized by aberrant cell cycle activities, which occur either as a result of mutations in upstream signaling pathways or by genetic lesions in genes encoding cell cycle proteins [56]. Thus, cell cycle regulators are attractive targets, leading to recent FDA approval of several CDK4/6 inhibitors for human cancers [57,58,59,60,61]. However, frequent inactivation of RB1 by loss of function mutation or MCPyV enables MCC proliferation independent of CDK4/6 activity, making CDK4/6 inhibitors less effective in MCC. Moreover, mutations in PI3K and its downstream molecules AKT and mTOR have been detected in more than 50% of MCC tumors, and activation of the PI3K/mTOR pathway is observed in up to 80% of all MCCs, subverting the normal requirement for cell proliferation [16,17,18,47,62]. In an effort to identify additional therapeutic targets, we performed high throughput drug screening against >1500 clinically relevant compounds with a panel of six MCC cell lines and identified AURK as a promising target in MCC.

Although AURKA, AURKB, and AURKC are highly homologous, they have distinct functions. AURKA is essential for centrosome maturation, spindle assembly, and spindle orientation. Moreover, AURKA phosphorylates PLK1, thereby promoting CDK1 activation and mitotic entry. AURKB controls chromosome alignment in mitosis and cytokinesis as a catalytic unit in the chromosome passenger complex [24]. AURKC expression is restricted to the testis and functionally mimics AURKB [63]. Analysis of human tumors supports oncogenic roles for AURKA and AURKB [64]. Overexpression of AURKA is detected in a wide spectrum of cancers, and it is associated with poor clinical outcomes, rendering it a highly important therapeutic target [25,26,51]. AURKA has been identified as a synthetic lethal target for several tumor suppressors, including *ARIDIA, SNF,* and *SMARCA4*, as well as *RB1* [54,65,66]. AURKA inhibitor synergizes with BET inhibitor against MYCN-positive human glioblastoma [41]. Moreover, up-regulation of AURKA/PLK/CDK1 contributes to PI3K inhibitor resistance in glioblastoma [67], and AURKA drives the evolution of resistance to EGFR inhibitor in lung cancer [31].

Inhibition of AURKA is superior because AURKB-inhibition causes DNA endoreduplication and polyploidy and can potentially increase the refractory and resistant cell population [36]. While several AURK inhibitors have been developed over the years, they either lack potency or high selectivity for AURKA inhibition [38]. Recently developed highly-selective AURKA inhibitor, AK-01, has demonstrated over 1000-fold selectivity for AURKA versus AURKB, leading to mitotic arrest and apoptosis across many human cell lines and in animal models. Likewise, AK-01 exhibited anti-tumor activities in MCC cells by cell cycle arrest and inducing apoptosis. Recently, it has been reported that AK-01 confers unique sensitivity in tumors without RB expression [43,51]. Similarly, the low IC_50_ in two MCPyV-negative MCC-3 and MCC-9 cells correlates with a lack of RB expression, respectively (Figure 1 and Figure S1). In MCC-9 cells without RB expression, AK-01 induces G2-M cell cycle arrest as suggested by the accumulation of cyclin B and histone three (Figure 2 and Figure 3). Increased apoptosis is also observed upon treatment, as evident by increased MCC-9 cells in the sub-G1 and elevated cleaved PARP, as demonstrated by immunoblotting (Figure 2). This corroborates our in vivo observation that AK-01 significantly suppresses MCC xenograft tumor growth during treatment (Figure 4). Moreover, AK-01 has more potent anti-tumor activities in MCC-9 xenografts, which is consistent with the notion that AURKA inhibitor retained synthetic lethality in cancer cells with RB1 loss in lung cancers [43]. In contrast, RB expression was detected in all 3 MCPyV-positive cell lines (Figure S1). Interestingly, even with RB expression, MKL-1 cells are more responsive to AK-01 treatment as compared to MCC-21 and MCC-16 cells, resulting in the regression of MKL-1 xenografts upon AK-01 treatment. Therefore, we argue that AK-01 leads to distinct cytotoxic effects on MCC cells harboring MCPyV (Figure 1 and Figure S1), justifying further investigation.

AURKA has been shown to phosphorylate AKT and mTOR in promoting chemotherapy resistance [40]. This is unlikely the underlying mechanism leading to increased apoptosis in MCC cells as p-AKT and p-mTOR levels were unchanged upon AK-01 treatment. As AURKA inhibitors have demonstrated anti-tumor activities either as a single agent or in combination in *MYCN*-positive human glioblastoma and AURKs are attractive therapeutic targets in *c-Myc*-driven lymphoproliferative disorders [32]. However, examination of mRNA expression failed to reveal a definitive association between *Myc* expressions upon AK-01 treatment in MCC cells and xenografts.

Recently, AK-01 has demonstrated activities in patients with tolerable side effects in an early-stage clinical trial [44]. However, one of the enduring challenges in cancer management is the resistance developed to targeted therapy. Surprisingly, we observe the short durability after discontinuation of AK-01 treatment in both MCC-9 and MKL-1 xenografts. We reason that AK-01 might be less effective to non-cycling cancer stem cells, and AK-01 might be advantageous in the combinatorial setting. Although, in the study by Gong et al., which demonstrates a response to AK-01 after relapse in the ovarian cancer models, MKL-1 xenografts have developed resistance and fail to retain xenograft tumor growth upon re-treatment [43]. Ideally, in vivo testing should be performed in an immune-competent model system; however, the lack of syngeneic animal models of MCC has hampered preclinical studies. Collectively, our studies have shown the vulnerability of MCC to AURKA inhibition by AK-01 and that AK-01 inhibition confers synthetic lethality in MCC cells with RB loss. Interestingly, our data infer that the cytotoxic effects of AURKA inhibition by AK-01 on MCPyV-positive MCC are independent of RB expression and deserve further investigation. Finally, the swift response and resistance development after short-term exposure to AURKA inhibition provide an important preclinical observation, highlighting the promises and obstacles in utilizing AURK inhibitors in MCC management.

## 5. Conclusions

Using patient-derived genetic insights as well as high-throughput drug screening, we have identified aurora kinase as a therapeutic target in MCC. Our current study provides conclusive evidence supporting LY3295668 (AK-01), a highly AURKA-selective drug, as a promising candidate for MCC management. Consistent with published studies, our data associates this anti-MCC activity of AK-01 to its ability to induce G2-M cell cycle arrest and apoptosis in an RB-dependent manner in MCPyV-negative MCC. Consistent with these results, AK-01 profoundly debilitated xenograft tumor growth. Interestingly, discontinuation of AK-01 resulted in swift relapse with accrued resistance. Our results support AK-01 as a potential candidate drug for combating MCC and provide critical evidence for its clinical translation.

## Data Availability

The data presented in this study are available on request from the corresponding author.

## Supplementary Materials

The following are available online at https://www.mdpi.com/article/10.3390/cancers13153708/s1, Figure S1: Basal level of Rb1 expression in MCC cells, Figure S2: Body weight of mice receiving AK-01 treatment, Figures S3–S6: Full western blot images for Figure 2, Figure S7: Full western blot image for Figure S1, Table S1: Evaluation of AK-01 toxicity in mice.
