# Supplementary material for: Selective Inhibition of Aurora Kinase A by AK-01/LY3295668 Attenuates MCC Tumor Growth by Inducing MCC Cell Cycle Arrest and Apoptosis
Source: Cancers (Basel). 2021 Jul 23;13(15):3708. doi: 10.3390/cancers13153708 (PMC8345130; doi:10.3390/cancers13153708)
Supplement: Supplementary file 1 [file cancers-13-03708-s001.zip › cancers-1302648-supplementary.pdf]

Supplementary Materials

Selective Inhibition of Aurora Kinase A by AK-01/LY3295668 Attenuates MCC Tumor Growth by Inducing MCC Cell Cycle Arrest and Apoptosis

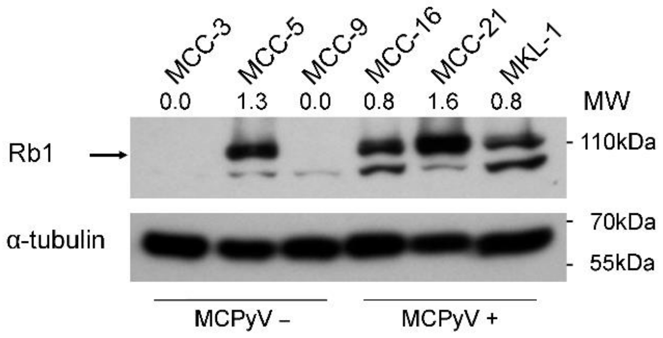

Figure S1. Basal level of Rb1 expression in MCC cells. MCC cells were harvested and 10–20 micrograms of total cell lysate per sample was resolved in 10% SDS PAGE followed by immunoblotting against Rb1 specific antibody. Total tubulin was used as loading control. Densitometry data (ratio of Rb1 compared to tubulin) was calculated using ImageJ and is presented above each lane. Full western blot image is available in Figure S7. MW, molecular weight; kDA, kilodaltons.

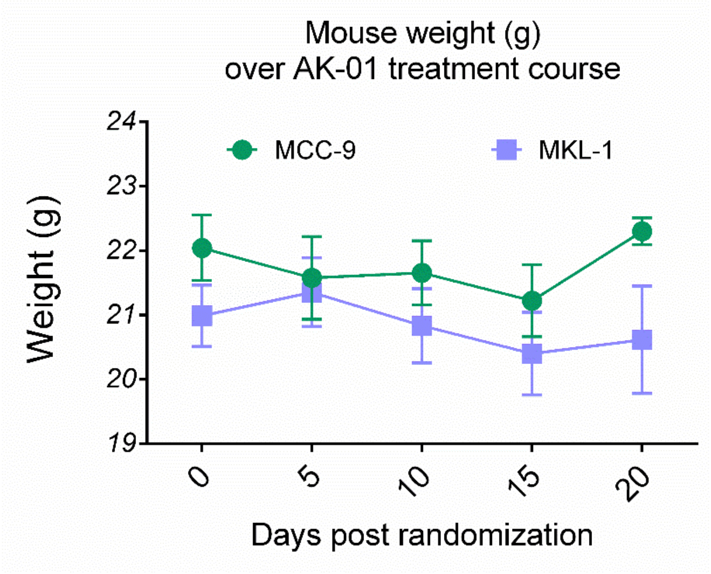

Figure S2. Body weight of MCC-bearing mice receiving AK-01 treatment.

Table S1. Evaluation of AK-01 toxicity in MCC-bearing mice.

	Posture	Activity	Body Condition Score	Food Intake	General Appearance
**MCC-9 cohort** (*n* = **9**)	Normal (9 of 9)	Alert, active (9 of 9)	BCS 3, well-conditioned (9 of 9)	Normal appetite & feces (9 of 9)	Normal, well groomed (9 of 9)
**MKL-1 cohort** (*n* = **9**)	Normal (9 of 9)	Alert, active (9 of 9)	BCS 3, well-conditioned (9 of 9)	Normal appetite & feces (9 of 9)	Normal, well groomed (9 of 9)

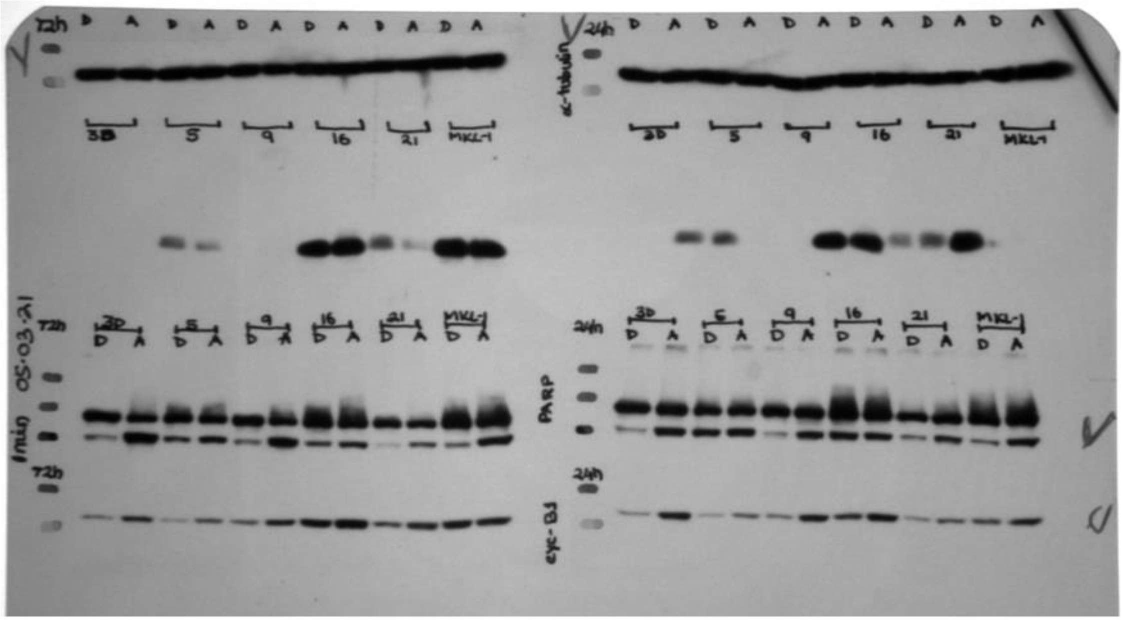

Figure S3. Full western blot image for Figure 2 of PARP, cycB1, and tubulin expression for MCC cells.

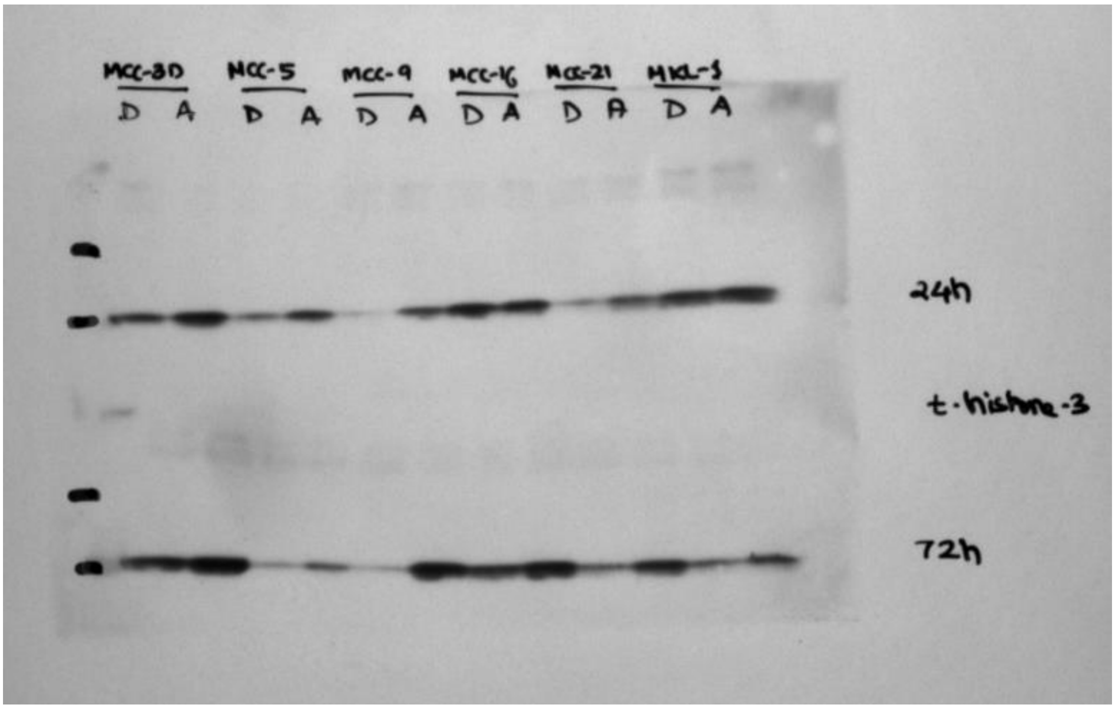

Figure S4. Full western blot image for Figure 2 of t-histone-3 expression for MCC cells.

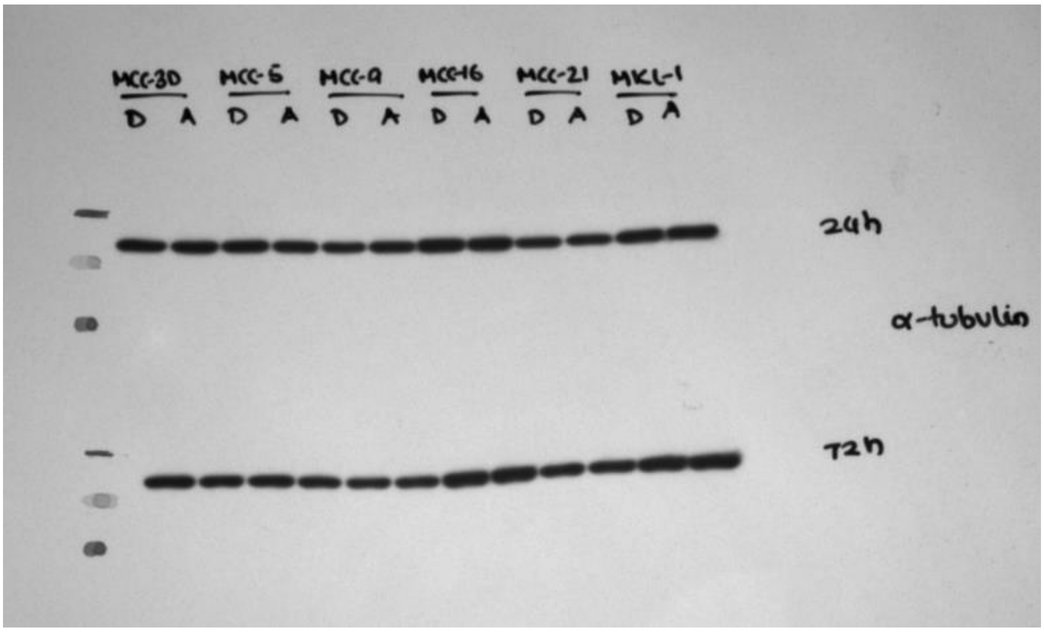

Figure S5. Full western blot image for Figure 2. of tubulin expression for MCC cells.

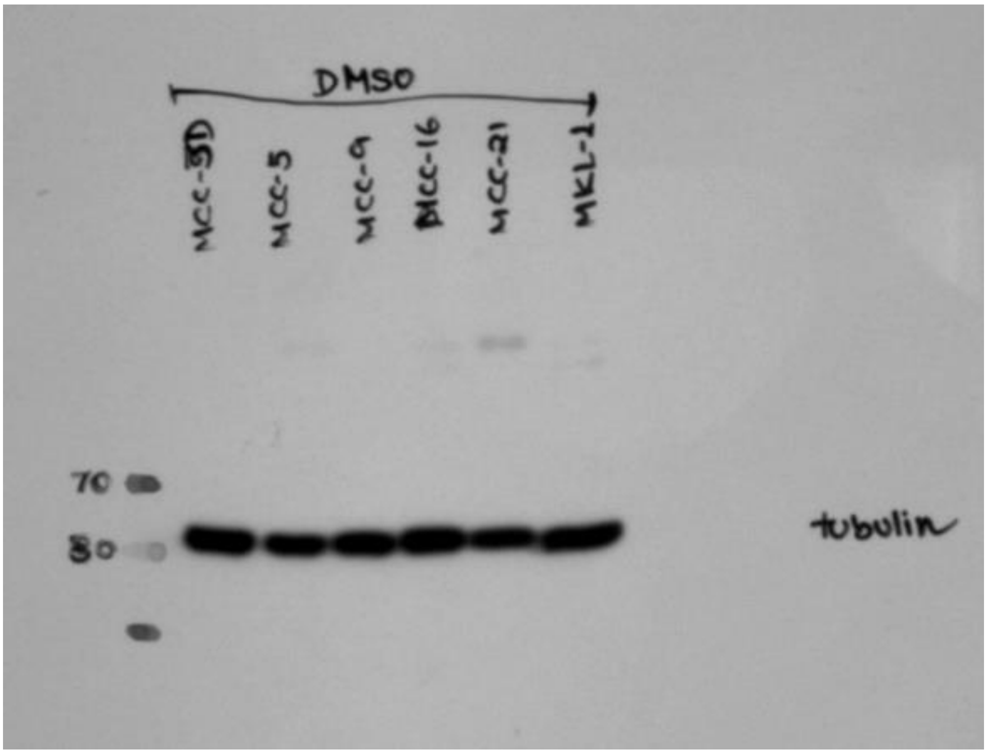

Figure S6. Full western blot image for Figure 2. of tubulin expression for MCC cells in DMSO.

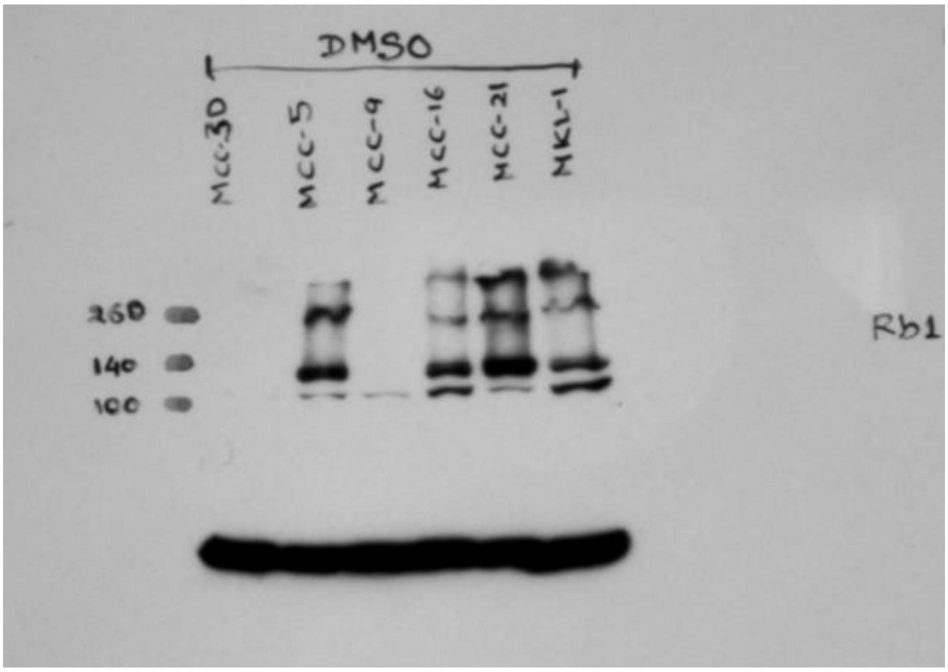

Figure S7. Full western blot image for Figure S1 of Rb1 expression for MCC cells in DMSO.
